# The Sinking Heart: A case of left anterior descending artery intervention complicated by perforation and cardiac tamponade

**DOI:** 10.21542/gcsp.2020.35

**Published:** 2020-12-31

**Authors:** Mladen Grigorov, Mohammad Mathbout, Ibrahim Fahsah, Shahab Ghafghazi

**Affiliations:** 1 Department of Internal Medicine, University of Louisville School of Medicine, Louisville, Kentucky, 40202; 2 Division of Cardiovascular Medicine, Department of Medicine, University of Louisville School of Medicine, Louisville, Kentucky, 40202; 3 Division of Cardiovascular Medicine, Department of Medicine, Norton Heart & Vascular Institute, Louisville, Kentucky, 40202

## Abstract

Coronary interventions are one of the most commonly performed procedures in interventional medicine. They have provided a life-prolonging and -saving solution, but are not without their own complications. These, although rare, do occur and are important to recognize in order to promptly and efficiently provide a solution to prevent catastrophic consequences to the patient.

We present a 70-year-old male with a past medical history significant for hypertension, hyperlipidemia, and myasthenia gravis; who presented to the hospital with substernal, pressure-like chest pain with associated nausea and diaphoresis. He was found to have ST segment elevations in anterolateral leads, prompting catherization lab activation revealing left anterior descending (LAD) artery stenosis.

Percutaneous intervention via balloon dilation and stent placement was performed with periprocedural mid-intervention hemodynamic collapse occurring. Subsequent left ventricular (LV) angiography was performed revealing preserved LV function without perforation - however a rim of contrast was noted surrounding the LV. Thus, hemodynamic collapse was recognized as result of cardiac tamponade with pericardial drain emergently inserted resulting in hemodynamic recovery. Our case aims to present a case of vascular perforation with the uniqueness in our diagnostic approach via fluoroscopic imaging.

## Case report

**Figure 1.**
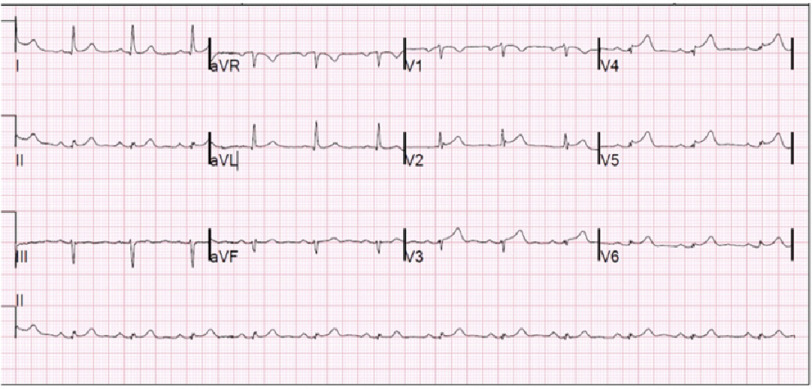
EKG from initial presentation.

We present a 70-year-old male with a past medical history significant for hypertension, hyperlipidemia, and myasthenia gravis; who presented to the hospital with substernal, pressure-like chest pain, with associated nausea and diaphoresis. EKG was obtained showing ST segment elevations in anterolateral leads (Figure 1), prompting acute coronary syndrome protocol and catherization lab activation.

**Figure 2.**
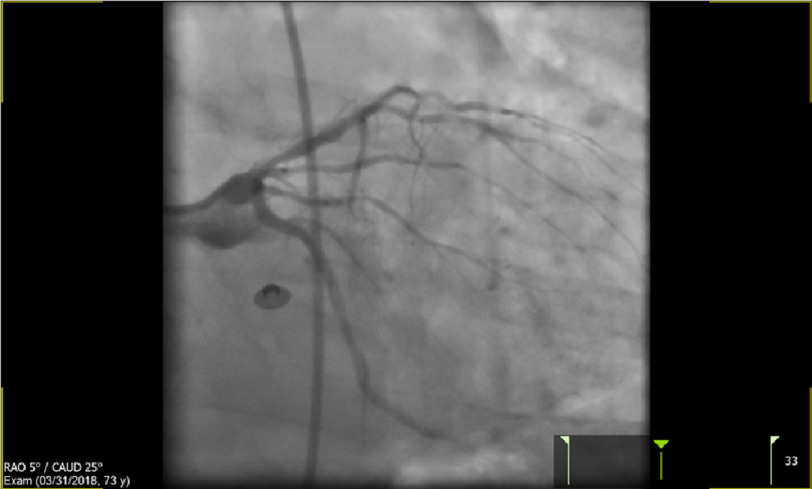
Showing moderate-severe LAD stenosis in the patient.

During catherization, coronary angiography revealed a moderate to severe stenotic segment in the proximal LAD artery (Figure 2), prompting advancement of an interventional wire across the stenotic segment, pre-dilation with a 3.0 × 15 NC balloon, and placement of a 3.5 × 28 mm Xience stent. Subsequent LAD angiography initially appeared fine.

Shortly afterwards the patient started to exhibit hemodynamic collapse, with a drop of blood pressure to 60/40s and heart rate in the 140s. Emergent search for the etiology of hemodynamic instability was undertaken. This consisted of LV angiography ruling out perforation as well as re-examination via angiography of the right coronary artery (RCA), left anterior descending artery (LAD), and left circumflex artery (LCx) ruling out vascular occlusion.

**Figure 3.**
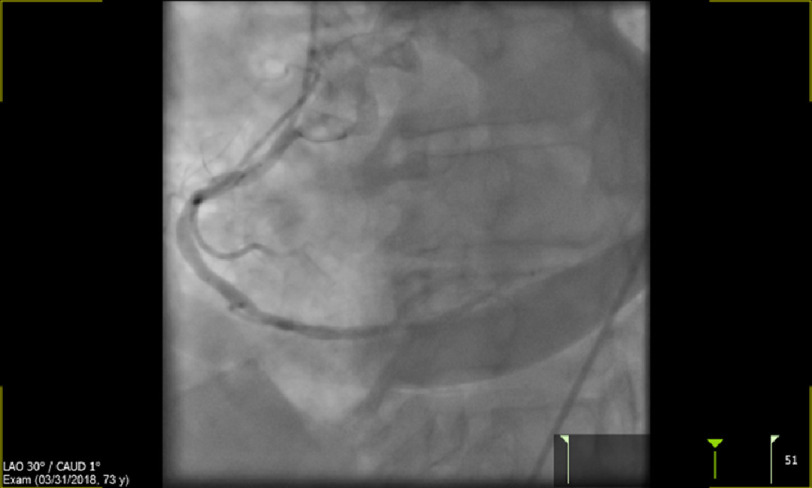
Left ventricular contrast rim.

**Figure 4.**
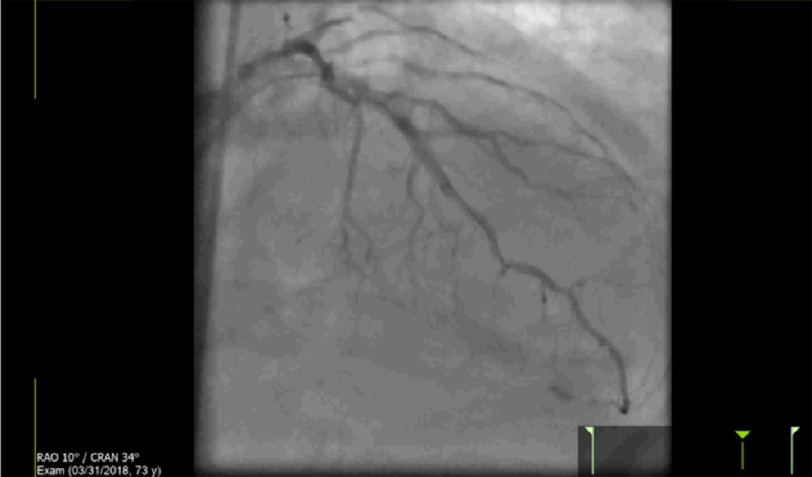
Distal LAD perforation.

**Figure 5.**
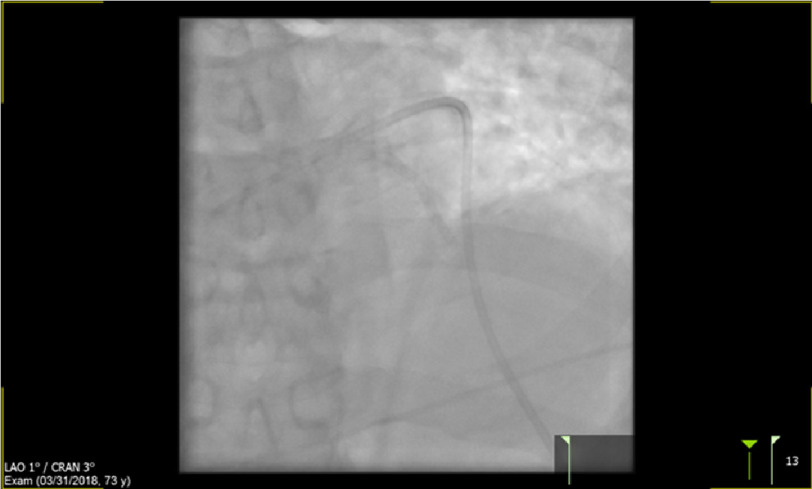
Pericardial drain inserted.

However, a rim of contrast surrounding the LV was noted (Figure 3). As a result of this, repeat LAD angiography was quickly performed which revealed distal vessel perforation and extravasation of contrast (Figure 4). Thus, the etiology of the patient’s hemodynamic instability was attributed to the coronary wire causing artery perforation, resulting in cardiac tamponade. A pericardial drain was emergently inserted over a wire with subsequent hemodynamic recovery of patient (Figure 5).

## Discussion

Coronary interventions are not without their own complications. These include myocardial infarction, cerebrovascular accident, arrythmia, vascular complications, contrast allergic reactions, perforation of heart chamber, as well as death. Perforation of coronary arteries occur in the setting of trauma caused by guidewires, atherectomy devices, balloon dilations and even stent placement ^1–4^. It has been reported at a rate ranging from 0.2–0.6% of patients undergoing PTCA with the reported risk being on the higher side with the use of athero-ablative devices ^5,6^.

Although rare, all of these run the risk of causing damage to the native vessel leading to perforation with potential catastrophic consequences such as cardiac tamponade^7,8^. Time is not an interventionalist’s ally in presentations such as these and thus a broad differential with prompt rule outs needs to be performed.

In our case, upon rapid recognition of patient’s hemodynamic compromise, we proceeded with a general breakdown of etiology categories into arrhythmogenic and mechanical (e.g., free wall rupture, severe left ventricular dysfunction, ventricular septal defect, acute valvular dysfunction, stent re-occlusion/vessel thrombosis, vascular perforation leading to tamponade).

The consideration of a broad differential is important as respective management for resolution would differ for each. For instance, free wall rupture management is surgical, but often a fatal complication^9^ whereas severe left ventricular systolic dysfunction requires a left ventricular assist device (LVAD)^10,11^. Ultimately, the various potential complications and their differing management plans have been very well reported in the respective literature^12^.

Thus, in order to accurately define and manage our presentation we began systematically ruling out differentials. Arrhythmogenic was ruled out via telemetry and rhythm strips being remarkable mainly for sinus rhythm in 140s with occasional NSVT episodes. Following this we ruled out left ventricular dysfunction, ventricular septal defect, and free wall rupture via left ventricular angiogram showing symmetrical muscle contraction, no wall perforation, or any significant valvular dysfunction. We then repeated vessel examinations (RCA/LAD/LCx) via angiography ruling out vascular occlusion but the fluoroscopy revealed a rim of contrast around the left ventricle.

This prompted re-examination via arteriogram again where distal LAD perforation was noted with contrast extravasation. Subsequent management of artery perforation consistent of localized balloon inflation and reversal of anticoagulation to promote closure via hemostasis at injury site as well as emergent insertion of a pericardial drain to remove pericardial effusion allowing for hemodynamic recovery in our patient.

## Conclusion

Coronary artery perforation is very rare with fewer than 1% of cases noted as presented. It can stem from the result of trauma caused by guidewires, atherectomy devices, balloon dilations and even stent placement. Fortunately, its overall incidence continues to decline with technological advances, but although rare it does still occur - thus, it should always be at the forefront of a provider’s mind when performing these procedures. We hope that through continued spotlighting, physicians will quickly and effectively rule out a broad differential and recognize the complication in order to promptly provide its respective therapy that can result in a life-saving resolution.
